# Integrative Metabolomics, Pharmacoinformatics and Experimental Studies Reveal the Neuroprotective Potential of *Caulerpa racemosa* Metabolites Against Alzheimer’s Disease

**DOI:** 10.3390/md23120475

**Published:** 2025-12-11

**Authors:** Nita Handayani, Dhecella Winy Cintya Ningrum, Adha Fauzi Hendrawan, Anis Yuniati, Raffaele Romano, Lucia De Luca, Antonello Santini, Fahrul Nurkolis

**Affiliations:** 1 Department of Physics, Faculty of Science and Technology, Universitas Islam Negeri (UIN) Sunan Kalijaga Yogyakarta, Yogyakarta 55281, Indonesia; 2 Master’s Program in Pharmaceutical Science, Faculty of Pharmacy, Universitas Gadjah Mada, Yogyakarta 55281, Indonesia; 3 Department of Medicine, Faculty of Medicine, Public Health, and Nursing, Universitas Gadjah Mada, Yogyakarta 55281, Indonesia; adhafauzihendrawan2004@mail.ugm.ac.id; 4 Department of Agricultural Sciences, University of Napoli Federico II, Piazza Carlo di Borbone 1, 80055 Portici, NA, Italy; 5 Department of Pharmacy, University of Napoli Federico II, Via Domenico Montesano 49, 80131 Napoli, Italy; 6 Master of Basic Medical Science, Faculty of Medicine, Universitas Airlangga, Surabaya 60286, Indonesia; fahrul.nurkolis.mail@gmail.com; 7 Medical Research Center of Indonesia, Surabaya 60286, Indonesia; 8 Institute for Research and Community Service, State Islamic University of Sunan Kalijaga (UIN Sunan Kalijaga), Yogyakarta 55281, Indonesia

**Keywords:** *Caulerpa racemosa*, Alzheimer’s disease, metabolomics, network pharmacology, molecular docking, acetylcholinesterase, nitric oxide synthase, neuroinflammation, antioxidant activity, marine functional food

## Abstract

Alzheimer’s disease (AD) is a multifactorial neurodegenerative disorder characterized by cholinergic dysfunction, oxidative/nitrosative stress, and neuroinflammation. Marine green algae *Caulerpa racemosa* are rich in neuroactive lipids and fatty acid derivatives with reported antioxidant and anti-inflammatory properties. However, their integrated mechanistic potential against AD remains largely underexplored. This study aimed to elucidate the neuroprotective mechanisms of *C. racemosa* metabolites against AD using integrative metabolomics, network pharmacology, molecular docking, and in vitro validation assays. Untargeted LC–HRMS profiling was performed to identify major metabolites in the ethanolic extract of *C. racemosa*. Neuroprotective targets were predicted via TargetNet, STRING, and Cytoscape (MCODE, CytoNCA). Functional enrichment was conducted using KEGG, GO (BP, MF, CC), and ClueGO. Molecular docking (CB-Dock2) validated compound–target interactions with ACHE, CHRM1, NOS1, and NOS2. Antioxidant (DPPH) and cholinesterase (AChE/BChE) inhibitory activities were evaluated in vitro. Metabolomic profiling identified lipid-dominant metabolites—oleamide, hexadecanamide, palmitoyl ethanolamide, α-linolenic acid, α-eleostearic acid, and 9-oxo-octadecadienoic acid. Network analysis revealed key AD-related hubs (ACHE, CHRM1, NOS1, NOS2) enriched in cholinergic regulation, arachidonic-acid metabolism, oxidative stress response, and nitric oxide signaling. Docking showed moderate multi-target affinities (−6.0 to −8.4 kcal/mol), with α-linolenic acid, α-eleostearic acid, and oxidized C18 lipids exhibiting the strongest interactions—particularly with ACHE and NOS isoforms. In vitro assays showed moderate antioxidant activity (IC_50_ = 120.97 ± 10.93 µg/mL) and cholinesterase inhibition (AChE IC_50_ = 136.48 ± 1.70 µg/mL; BChE IC_50_ = 145.98 ± 3.28 µg/mL), aligning with predicted multi-target interactions. *C. racemosa* extract exhibits neuroprotective potential through a synergistic combination of cholinergic modulation, antioxidant activity, NOS-mediated nitrosative stress reduction, and suppression of arachidonic-acid inflammatory pathways. These findings support *C. racemosa* as a promising marine-derived multi-target candidate for AD intervention, warranting further mechanistic and in vivo evaluation.

## 1. Introduction

Alzheimer’s disease (AD) represents one of the most devastating neurodegenerative disorders, characterized by progressive cognitive decline and neuronal loss driven by oxidative stress, amyloid-β aggregation, cholinergic dysfunction, and neuroinflammation [1,2]. Despite decades of research, currently available treatments such as donepezil, rivastigmine, and memantine only provide symptomatic relief rather than halting disease progression [3]. Therefore, the search for novel, multi-targeted, and naturally derived neuroprotective agents has gained increasing momentum. Among natural sources, marine algae have emerged as prolific reservoirs of bioactive metabolites with diverse pharmacological properties relevant to AD therapy, including antioxidant, anti-inflammatory, and acetylcholinesterase inhibitory activities [4,5].

Marine green algae, particularly *Caulerpa racemosa* (sea grapes), are rich in bioactive lipids, fatty acids, and amides such as oleamide, hexadecanamide, and α-linolenic acid [6]. These metabolites have been associated with neuronal protection through modulation of cholinergic transmission, nitric oxide synthase (NOS) pathways, and synaptic plasticity [7]. Recent advances in marine pharmacology highlight that marine-derived fatty acids and amides can simultaneously act on acetylcholinesterase (AChE), muscarinic receptors (CHRM1), and nitric oxide synthase isoforms (NOS1/NOS2), offering a multi-target approach to combat AD pathology [8]. In particular, oleamide and α-linolenic acid, both of which are found in marine algae, have shown promising inhibitory effects against cholinesterase enzymes and attenuation of neuroinflammation, aligning with findings from recent studies in *Marine Drugs* emphasizing marine algal metabolites as multifunctional agents in AD management [9].

Integrative metabolomics combined with network pharmacology and molecular docking has become a powerful strategy to elucidate bioactive compound–target interactions within complex biological systems [10]. Such approaches enable the identification of key molecular targets and pathways mediating neuroprotection. In this study, we employed LC–HRMS-based metabolomic profiling of *C. racemosa* extract, followed by in silico network pharmacology and molecular docking to predict its interaction with AD-related targets. Furthermore, in vitro assays were performed to validate its antioxidant and cholinesterase inhibitory potential. This integrative approach offers novel insights into the mechanistic landscape of *C. racemosa* as a marine-derived candidate for the prevention or adjunctive management of Alzheimer’s disease.

## 2. Results

### 2.1. Metabolomic Profiling of Caulerpa Racemosa Extract

Untargeted LC–HRMS analysis of the *Caulerpa racemosa* ethanolic extract revealed a diverse metabolite profile dominated by bioactive fatty acid amides and unsaturated fatty acids (Table 1). Nine major compounds were identified with high confidence scores (mzCloud ≥ 95%), including hexadecanamide, oleamide, stearamide, α-eleostearic acid, palmitoleic acid, 9-oxo-10(E),12(E)-octadecadienoic acid, α-linolenic acid, palmitoyl ethanolamide, and 2,4-dihydroxyheptadec-16-en-1-yl acetate. Among these, *oleamide* and *α-linolenic acid* have been previously reported as neuroactive lipids with known roles in modulating sleep–wake cycles, cholinergic transmission, and anti-inflammatory responses. The retention times of 16–24 min and molecular weights ranging from 254 to 350 Da suggest these compounds belong to long-chain lipids with potential to cross the blood–brain barrier, a critical pharmacokinetic feature for neuroprotective agents. The strong match with known MS/MS spectral libraries supports their reliability for downstream pharmacoinformatic analyses.

### 2.2. Network Pharmacology and Protein–Protein Interaction

**Figure 1:**
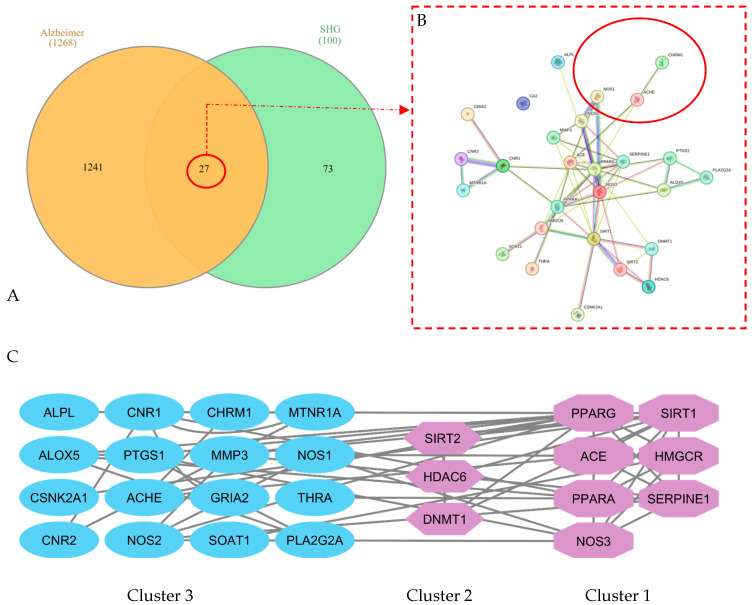
Venn diagram analysis (**A**); protein–protein interaction of potential targets of compounds in Alzheimer’s (**B**); clustering by molecular complex detection (MCODE) (**C**). SHG: Sea grapes extract compounds.

To elucidate the molecular targets associated with Alzheimer’s disease (AD), overlapping gene sets between *C. racemosa* metabolites and AD-related genes were analyzed using Venn diagram mapping (Figure 1A). The intersection revealed a substantial overlap of neurodegeneration-related targets, indicating that several metabolites potentially modulate core signaling pathways involved in AD pathology. The protein–protein interaction (PPI) network constructed through the STRING database (Figure 1B) displayed a highly interconnected topology, highlighting key nodes such as ACHE, CHRM1, NOS1, and NOS2. These proteins are central to cholinergic neurotransmission and nitric oxide signaling, two pathways known to deteriorate during AD progression. Cluster analysis using the MCODE algorithm (Figure 1C) identified three major functional modules, suggesting that the active compounds from *C. racemosa* may simultaneously regulate oxidative stress responses, neurotransmitter balance, and inflammatory cascades.

### 2.3. Functional Enrichment and Biological Process Clustering

**Figure 2:**
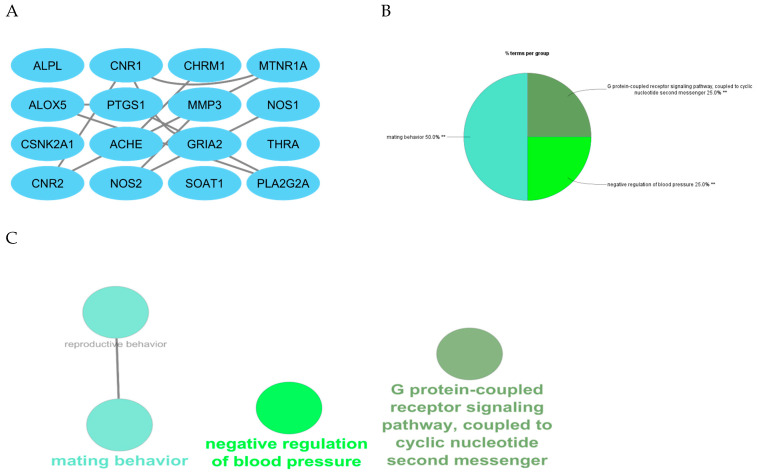
Chosen cluster (cluster of 3) (**A**), Percentage of terms of biological process (**B**), and Subcluster of biological process genes (**C**).

Cluster 3, identified as the most biologically significant, was further analyzed using ClueGO for Gene Ontology (GO) enrichment (Figure 2A–C). The results demonstrated that the predominant biological processes included response to oxidative stress, synaptic signaling, nitric oxide biosynthesis, and acetylcholine catabolic regulation. The clustering of sub-biological processes further reinforced the hypothesis that *C. racemosa* metabolites act through multi-targeted mechanisms, particularly modulating enzymes associated with neurotransmitter degradation (ACHE/BChE) and neuroinflammation (NOS1/NOS2). The distribution of functional terms underscores the integrative mode of action characteristic of natural compounds compared to single-target drugs.

**Figure 3:**
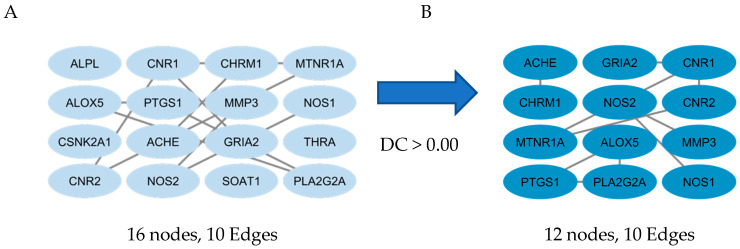
Interprotein interaction using CytoNCA, screening proteins based on degree centrality (DC) score threshold > 0.00. (**A**) the initial protein–protein interaction network before filtering, and (**B**) the filtered core network based on the degree centrality (DC) threshold.

Building on the ClueGO biological-process results above, the PPI analysis in Figure 3 shows that these functions are supported by a tightly connected target core. The initial network (16 nodes, 10 edges) was refined by degree centrality (DC > 0.00) to 12 highly connected hubs without losing edges, indicating stable interprotein cooperation. Key nodes ACHE, NOS1/NOS2, PTGS1, ALOX5, PLA2G2A, GRIA2, CHRM1, CNR1/CNR2, MTNR1A, and MMP3—suggest a multi-target mode where cholinergic degradation (ACHE), nitric oxide–linked oxidative/nitrosative stress (NOS1/NOS2), and arachidonic-acid neuroinflammation (PTGS1/ALOX5/PLA2G2A) converge with synaptic signaling receptors (GRIA2, CHRM1) and neuromodulators, reinforcing the earlier hypothesis of integrated neuroprotective control.

**Figure 4:**
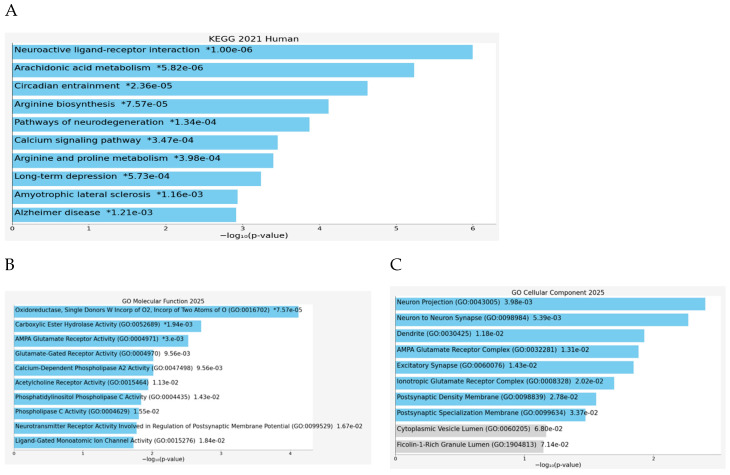
Enrichment analysis of key target proteins, KEGG pathway analysis (**A**); Molecular Function (**B**); and Cellular Components (**C**).

Consistent with this hub architecture, Figure 4A (KEGG) highlights neuroactive ligand–receptor interaction and arachidonic acid metabolism as top pathways, with additional enrichment in circadian entrainment, calcium signaling, long-term depression, and neurodegeneration routes (e.g., Alzheimer’s/ALS), indicating simultaneous synaptic and inflammatory regulation. Figure 4B (GO Molecular Function) further specifies the mechanisms as oxidoreductase and nitric-oxide–related activities, carboxylic ester hydrolase/cholinesterase functions, phospholipase A2 activity, and glutamate/acetylcholine receptor activities, matching the main hubs from Figure 3. Figure 4C (GO Cellular Component) localizes these targets to neuron projections, dendrites, synapses, AMPA receptor complexes, and postsynaptic density, showing that *C. racemosa* metabolites likely act at excitatory synaptic sites where oxidative stress and neuroinflammation shape plasticity, setting up the next section of results on how these pathway-level effects translate into therapeutic implications.

### 2.4. Molecular Docking Analysis

Molecular docking analysis is shown in Table 2, where it summarizes the docking affinities of *C. racemosa* metabolites against four Alzheimer’s-related targets (ACHE, CHRM1, NOS2, NOS1), where more negative Vina scores indicate stronger predicted binding. Overall, all tested compounds showed moderate interactions across the panel (−6.0 to −8.4 kcal/mol), supporting a multi-target profile consistent with the earlier network and enrichment findings. Among them, unsaturated fatty acids and oxidized lipid derivatives tended to perform better than simple amides, suggesting that double bonds and oxygenated groups may enhance target complementarity within both neurotransmission and oxidative-inflammatory proteins.

Target-wise, the strongest natural-compound binding was observed for ACHE, led by α-linolenic acid (−8.4), α-eleostearic acid (−8.3), and 9-oxo-octadecadienoic acid (−8.1), indicating notable potential to interfere with cholinesterase activity. For CHRM1, the best affinity was shown by 9-oxo-octadecadienoic acid (−7.4) and α-linolenic acid (−7.2), aligning with synaptic receptor modulation inferred from Figure 4. In the oxidative-stress axis, α-eleostearic acid displayed the strongest binding to NOS2 (−7.5), while α-eleostearic acid and α-linolenic acid were again highest for NOS1 (both −7.0/−6.7 range), reinforcing their relevance to nitric-oxide-related neuroinflammation. Importantly, the control drug donepezil showed substantially stronger docking across all targets (e.g., −11.8 for ACHE), which is expected for a single-target optimized pharmaceutical; however, the natural compounds’ consistent mid-range affinities across multiple proteins support the proposed broad, synergistic mechanism rather than a one-protein dominant effect.

Table 3 shows that α-linolenic acid binds along a hydrophobic channel in the ACHE active gorge, stabilized mainly by van der Waals/hydrophobic contacts with residues such as Trp286, Tyr337, Phe338, Tyr341, Phe297, Val294, and Ser293, and lying near the catalytic region (His447 and the Glu202/Ser203/Ala204 pocket), supporting its docking score in Table 2 and suggesting potential interference with substrate access. Donepezil occupies the same gorge more deeply and extensively, sharing key residues (Trp286, Tyr337, Tyr341, Phe297, Val294/Ser293, His447) but achieving tighter, broader interactions that explain its much stronger affinity. Overall, the residue overlap indicates a common binding corridor, while the simpler lipid scaffold of α-linolenic acid likely limits anchoring strength, reinforcing that *C. racemosa* metabolites may contribute moderate ACHE inhibition within a broader multi-target neuroprotective mechanism.

### 2.5. In Vitro Antioxidant and Cholinesterase Inhibitory Activities of C. racemosa Extract

Table 4 reports the IC_50_ values of the sea grape (*C. racemosa*) extract in antioxidant and cholinesterase assays, benchmarking against standard controls. The extract showed measurable but weaker activity than the references in all tests: DPPH scavenging IC_50_ was 120.97 ± 10.93 µg/mL versus Trolox at 99.04 ± 2.15 µg/mL, indicating moderate antioxidant capacity. For enzyme inhibition, the extract yielded IC_50_ values of 136.48 ± 1.70 µg/mL for AChE and 145.98 ± 3.28 µg/mL for BChE, whereas physostigmine was notably stronger (82.34 ± 1.45 and 83.81 ± 0.55 µg/mL, respectively). The superscript differences (a vs. b) suggest these reductions in potency are statistically significant.

When integrated with the in silico findings, these IC_50_ trends are expected, docking predicted mid-range affinities across multiple targets rather than drug-like single-target strength. Thus, the extract’s moderate cholinesterase inhibition and antioxidant effect likely reflect additive or synergistic contributions from several metabolites (e.g., fatty acids and amides) acting concurrently. This supports the broader mechanism proposed earlier—sea grape metabolites may not outperform classical inhibitors individually, but could provide neuroprotective benefit through combined anti-oxidative and multi-pathway cholinergic/anti-inflammatory modulation.

## 3. Discussion

**Figure 5:**
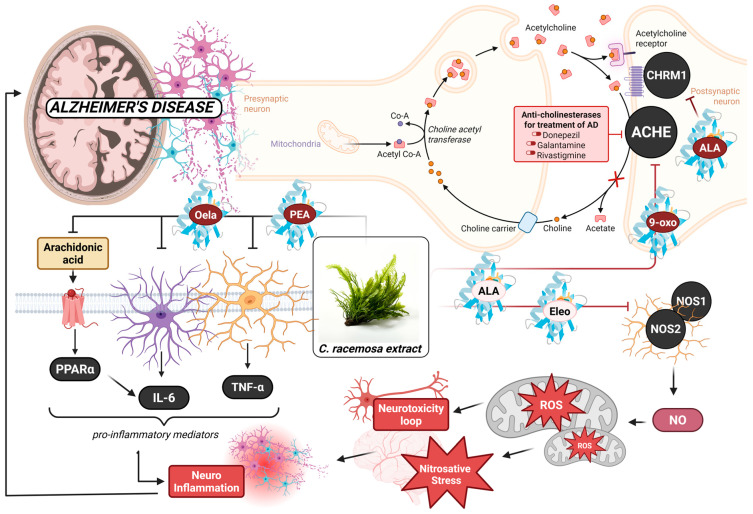
Proposed multi-target neuroprotective mechanisms of *Caulerpa racemosa* metabolites against Alzheimer’s disease. This integrative schematic illustrates how key metabolites identified in *C. racemosa*—including oleamide (Oela), palmitoyl ethanolamide (PEA), α-linolenic acid (ALA), α-eleostearic acid (Eleo), and 9-oxo-octadecadienoic acid (9-oxo)—interact with major Alzheimer’s disease (AD) pathological pathways. The metabolites demonstrate potential modulation of the cholinergic system by inhibiting acetylcholinesterase (ACHE) and supporting muscarinic acetylcholine receptor function (CHRM1), thereby enhancing acetylcholine availability at synapses. In parallel, the compounds exhibit predicted interactions with nitric oxide synthase isoforms (NOS1 and NOS2), reducing nitric oxide–mediated nitrosative stress and reactive oxygen species (ROS) generation. Additionally, *C. racemosa* lipids interfere with arachidonic-acid–driven pro-inflammatory cascades by influencing PPAR-α–linked signaling, suppressing IL-6 and TNF-α production, and attenuating neuroinflammation. Collectively, the multi-pathway interactions depicted in the figure support a synergistic mechanism in which cholinergic preservation, oxidative/nitrosative stress reduction, and lipid-mediated anti-inflammatory activity converge to mitigate neuronal damage and interrupt the neurotoxicity loop characteristic of AD progression. Created with BioRender.com license by Author (F.N.) (accessed on 10 October 2025).

The metabolomic profile of *Caulerpa racemosa* ethanolic extract in this study is dominated by fatty-acid amides (oleamide, hexadecanamide, stearamide) and unsaturated/oxidized fatty acids (α-linolenic acid, α-eleostearic acid, palmitoleic acid, 9-oxo-octadecadienoic acid), which is consistent with prior LC–HRMS and phytochemical works showing sea grapes as lipid-rich macroalgae with strong antioxidant-associated constituents [11]. Recent untargeted metabolomics on *C. racemosa* likewise reported broad lipid and secondary metabolite diversity, supporting the reliability of top-hit lipid signatures, while multiple antioxidant studies on *C. racemosa* extracts confirm that these chemical classes track with measurable radical-scavenging activity [12,13]. Taken together, our LC–HRMS findings extend previous chemical evidence by pinpointing specific neuroactive lipid candidates within the extract that are pharmacokinetically plausible for CNS action (Figure 5).

Network pharmacology and PPI analyses showed that the metabolites converge on ACHE, CHRM1, NOS1, and NOS2, with enrichment in neuroactive ligand receptor interaction, arachidonic-acid metabolism, oxidative-stress response, and synaptic signaling. This hub architecture mirrors established AD biology in which cholinergic breakdown co-evolves with nitric-oxide-related oxidative/nitrosative stress and lipid-driven neuroinflammation [14,15]. The presence of both cholinergic (ACHE/CHRM1) and redox-inflammatory (NOS1/NOS2; PTGS1/ALOX5/PLA2G2A) cores supports a multi-target strategy that has been repeatedly highlighted as more suitable for AD than single-node blockade, because synaptic loss is reinforced by feedback loops between ROS/RNS damage and microglia-mediated inflammation [16,17].

At the metabolite level, oleamide and palmitoyl ethanolamide (PEA) provide direct mechanistic bridges to earlier neurodegeneration research. Oleamide is a documented neuroactive fatty-acid amide that reverses scopolamine-induced cognitive impairment and restores cholinergic function, which aligns with its moderate docking to ACHE/CHRM1 in our dataset and the enrichment of synaptic receptor pathways [18]. PEA, an endogenous ALIAmide, has a proven ability to reduce reactive astrogliosis/microglial activation, suppress pro-inflammatory cytokines, and improve learning and memory in amyloid-based AD models via PPAR-α and related lipid-signaling targets [19,20,21]. Our identification of oleamide and PEA in *C. racemosa* suggests that sea-grape lipids may reproduce endogenous pro-homeostatic signaling that dampens neuroinflammation while supporting cholinergic tone.

The strongest docking affinities were observed for α-linolenic acid (ALA), α-eleostearic acid, and oxidized C18 derivatives, particularly toward ACHE and NOS isoforms, implying that unsaturation/oxygenation enhances complementarity to both neurotransmission and oxidative-stress proteins. This pattern is strongly supported by previous studies of ALA’s multi-axis protection in AD models, where ALA reduces Aβ-induced oxidative stress and nitric-oxide elevation, restores antioxidant defenses, attenuates neuroinflammation, and improves memory, while also regulating APP processing and neuronal apoptosis pathways [22,23]. Our findings demonstrate that ALA and donepezil share overlapping residue interactions within the AChE catalytic gorge, suggesting a partially common binding trajectory. The comparatively weaker anchoring of ALA, consistent with its lipid-based chemical scaffold, supports a mechanism of partial cholinesterase inhibition. This inhibitory profile may alleviate cholinergic symptoms while simultaneously permitting complementary disease-modifying activity mediated through NOS regulation and modulation of inflammatory lipid signaling pathways.

Finally, the in vitro data validate the system’s interpretation emerging from in silico results. The extract showed moderate antioxidant and AChE/BChE inhibitory activities lower than classical controls but consistent with predicted mid-range docking across multiple targets. Similar magnitudes of antioxidant capacity have been reported for *C. racemosa* extracts in earlier studies, reinforcing that sea-grape chemistry yields real redox buffering rather than purely computational promise [13]. When integrated, our findings support an AD-relevant mechanism where *C. racemosa* lipids (i) partially preserve acetylcholine signaling via ACHE/CHRM1 modulation, (ii) reduce oxidative/nitrosative stress through NOS1/NOS2 interaction and antioxidant reinforcement, and (iii) suppress arachidonic-acid and glial inflammatory cascades via PEA/oleamide-linked lipid mediators, collectively yielding a synergistic neuroprotective profile appropriate for the multifactorial pathology of Alzheimer’s disease.

## 4. Materials and Methods

### 4.1. Preparation of Green Algae Extract

The green algae (*Caulerpa racemosa*) were obtained from a cultivation pond located in Jepara Regency, Central Java, Indonesia, with authorization from the pond owner. The botanical identification and authentication of the species were verified at the State Islamic University of Sunan Kalijaga, Yogyakarta, Indonesia. The confirmation process employed the National Center for Biotechnology Information (NCBI) Taxonomy ID and the Integrated Taxonomic Information System (ITIS) Report ID, corresponding to *C. racemosa* (ID: 6968). Fresh biomass of *Caulerpa racemosa* was thoroughly washed with sterile seawater to remove impurities and epiphytic microorganisms. The cleaned material was then dried at 40 °C until the moisture content was reduced below 10%, followed by grinding into a fine powder. A total of 100 g of the dried powder was macerated with 1 L of 70% ethanol for 48 h at room temperature in a closed container, with occasional stirring to ensure optimal solvent–matrix interaction. After maceration, the mixture underwent ultrasonic-assisted extraction using a sonicator operating at 40 kHz for 30 min to enhance the solubilization of bioactive compounds. The filtrate was collected through Whatman No. 1 filter paper and concentrated under reduced pressure using a rotary evaporator at 45 °C to obtain a viscous greenish-brown extract [24].

### 4.2. Metabolomic Analysis

Untargeted metabolomic profiling of the sea grape extract was performed using liquid chromatography–high-resolution mass spectrometry (LC-HRMS) [25]. A 50 μL aliquot of the extract was diluted with 96% ethanol to a final volume of 1500 μL. The mixture was vortexed at 2000 rpm for 2 min, followed by centrifugation at 6000 rpm for 2 min. The resulting supernatant was filtered through a 0.22 μm syringe filter and transferred into an LC vial. The prepared sample was then placed in an autosampler for LC-HRMS analysis. The LC–HRMS system consisted of a Thermo Scientific Dionex Ultimate 3000 RSLCnano HPLC equipped with a microflow meter. The mobile phases used were: solvent A (0.1% formic acid in water) and solvent B (0.1% formic acid in acetonitrile). Chromatographic separation was achieved using a Hypersil™ GOLD aQ analytical column (50 × 1 mm, 1.9 μm particle size) with a flow rate of 40 μL/min and a total run time of 30 min. The column temperature was maintained at 30 °C. Mass spectrometric detection was conducted on a Thermo Scientific Q Exactive instrument operating in both positive and negative ionization modes. Full-scan spectra were acquired at a resolution of 70,000, with data-dependent MS/MS scans collected at 17,500 resolution for 30 min. Data processing and compound identification were carried out using Compound Discoverer software 3.4 integrated with the mzCloud MS/MS Library.

### 4.3. In Silico Analysis

#### 4.3.1. Identification of Potential Targets

The simplified molecular-input line-entry system (SMILES) codes of the investigated compounds were retrieved from the PubChem (https://pubchem.ncbi.nlm.nih.gov/, accessed on 17 September 2025) [26] and ChemSpider (https://www.chemspider.com/, accessed on 17 September 2025) [27] databases. Prediction of potential protein or gene targets was performed using the TargetNet platform (http://targetnet.scbdd.com/, accessed on 17 September 2025) [28], applying a probability threshold greater than 0.6 to ensure target reliability. To identify Alzheimer’s disease (AD)-related targets, the GeneCards (https://www.genecards.org/, accessed on 17 September 2025) and NCBI (https://www.ncbi.nlm.nih.gov/, accessed on 17 September 2025) [29] databases were queried using the keyword “Alzheimer’s.” The sets of compound-related and disease-related targets were compared using a Venn diagram to determine their intersection, representing the overlapping targets potentially involved in the pharmacological action of the compounds against AD [30].

#### 4.3.2. Network Analysis

The intersecting targets were imported into the STRING database (https://string-db.org/, accessed on 17 September 2025) [31] to evaluate protein–protein interactions (PPIs) and visualize their functional associations. The interaction network was then clustered using the MCODE plug-in in Cytoscape 3.10.3 to identify highly interconnected modules that may represent key biological complexes involved in AD pathology. Functional enrichment of each cluster was performed using the ClueGO plug-in, which integrates Gene Ontology (GO) and pathway information. Subsequently, the CytoNCA plug-in was applied to assess topological parameters, with Degree Centrality used as the selection criterion for hub genes. Targets with degree values greater than zero were subjected to Kyoto Encyclopedia of Genes and Genomes (KEGG) pathway and Gene Ontology analyses using the Enrichr database (https://maayanlab.cloud/Enrichr/, accessed on 17 September 2025) [32]. This analysis enabled the identification of genes significantly involved in AD-related biological processes and signaling pathways, which were subsequently selected for molecular docking validation [33].

#### 4.3.3. Molecular Docking Simulation

To validate the binding interaction between the bioactive compounds and the identified AD-related targets, molecular docking analysis was conducted. The three-dimensional crystal structures of the target proteins were obtained from the Protein Data Bank (PDB) (http://www.rcsb.org/pdb/home/home.do, accessed on 19 September 2025) [34]. The 3D structures of the selected compounds and target proteins were uploaded into the CB-DOCK2 web server (https://cadd.labshare.cn/cb-dock2/, accessed on 19 September 2025) [35], which performs automated docking based on cavity detection and binding site prediction. The docking outcomes were ranked according to binding affinity scores, with the lowest binding energy indicating the most stable and favorable ligand–receptor interaction. These results provided structural insights into the molecular mechanisms underlying the potential therapeutic action of the compounds against Alzheimer’s disease.

### 4.4. In Vitro Experiments

#### 4.4.1. Antioxidant Activity via DPPH Inhibition Assay

The antioxidant potential of green algae extracts was determined through DPPH radical scavenging, following the methods described by Nurkolis et al., 2023 [36]. In the DPPH assay, various concentrations of the green algae extract (35, 70, 105, 140, and 175 µg/mL) were mixed with 3 mL of DPPH solution in a testing vial. The mixture was then incubated for 30 min at room temperature under dark conditions. The reduction in DPPH radical concentration was quantified by measuring the absorbance at 517 nm.

The percentage of inhibition was calculated using the following equation:

(1) $$\%\;\mathrm{Inhibition}=100-\left(\frac{A0-A1}{A0}\right)\times 100\%$$

where A0 represents the absorbance of the control (blank), and A1 denotes the absorbance of the test sample or standard.

#### 4.4.2. AChE and BChE Inhibitory Assay

The in vitro inhibitory activities of the extract against acetylcholinesterase (AChE) and butyrylcholinesterase (BChE) were determined using a modified Ellman’s colorimetric method in a 96-well microplate format [37,38]. The assay is based on the enzymatic hydrolysis of acetylthiocholine iodide (ATCI) or butyrylthiocholine chloride (BTChCl) by AChE or BChE, respectively, releasing thiocholine, which reacts with 5,5′-dithiobis-(2-nitrobenzoic acid) (DTNB) to yield the yellow-colored 5-thio-2-nitrobenzoate anion detectable at 412 nm. The reaction mixture contained 140 µL of 0.1 M phosphate buffer (pH 8.0), 20 µL of 10 mM DTNB solution, and 20 µL of the test sample or standard inhibitor (physostigmine for AChE and rivastigmine for BChE). After gentle mixing, 10 µL of enzyme solution (AChE from *Electrophorus electricus* or BChE from equine serum, at a final activity of 0.02–0.06 U/mL) was added, followed by a 10 min pre-incubation at room temperature (25–28 °C) to allow reversible binding between the enzyme and inhibitor. The enzymatic reaction was initiated by adding 10 µL of 1.0 mM substrate solution (ATCI for AChE or BTChCl for BChE), resulting in a total volume of 200 µL per well.

The change in absorbance was recorded kinetically at λ = 412 nm every 30–60 s for 5–10 min using a microplate reader. Each assay was performed in triplicate. Blanks (without enzyme) and negative controls (without inhibitor) were included in each plate to correct for non-enzymatic background and baseline activity. The reaction rate (ΔA/min) for each well was calculated from the linear portion of the kinetic curve, and the percentage inhibition was determined using the equation:

(2) $$\%\;\mathrm{Inhibition}=100-\left(\frac{{\mathrm{slope}}_{\mathrm{sample}}}{{\mathrm{slope}}_{\mathrm{control}}}\times 100\right)$$

The concentration of the extract or compound that inhibited 50% of enzymatic activity (IC_50_) was obtained from nonlinear regression analysis of the concentration–response curve fitted to a four-parameter logistic (4PL) model. Selectivity between AChE and BChE inhibition was expressed as the Selectivity Index (SI = IC_50_ (BChE)/IC_50_(AChE)), where a value greater than 1 indicates preferential inhibition of AChE. All solutions were freshly prepared prior to the assay. The final DMSO concentration was kept below 0.5% *v/v* to prevent solvent-induced inhibition. For extracts with intense color or reducing properties, interference control wells containing sample, DTNB, and buffer (without enzyme and substrate) were included to correct background absorbance. The enzymatic activities were verified by reference inhibitors, with each plate’s positive control IC_50_ values remaining within the laboratory’s historical acceptance range, ensuring reproducibility and assay robustness.

### 4.5. Data Analysis and Management

All experimental results were obtained from at least three independent assays, each performed in triplicate to ensure reproducibility. Raw absorbance data were exported from the microplate reader and processed to obtain reaction rates (ΔA/min) through linear regression of absorbance versus time within the linear range of enzyme kinetics. The percentage inhibition at each concentration was calculated relative to the control group, and the IC_50_ values were determined by nonlinear regression fitting of the dose–response curves to a four-parameter logistic (4PL) model using GraphPad Prism version 10.6.1 (GraphPad Software, Boston, MA, USA).

The results were expressed as IC_50_ values (mean ± standard deviation, SD) with 95% confidence intervals (CI). Statistical comparisons between two independent groups (e.g., extract versus positive control, or between AChE and BChE inhibitory activity) were performed using the independent *t*-test after confirming data normality with the Shapiro–Wilk test and homogeneity of variances using Levene’s test. Differences were considered statistically significant at *p* < 0.05.

## 5. Conclusions

### 5.1. Conclusion

This study concludes that *Caulerpa racemosa* ethanolic extract contains a distinct lipid-dominated metabolite profile, primarily fatty acid amides and unsaturated/oxidized fatty acids such as oleamide, palmitoyl ethanolamide, α-linolenic acid, and α-eleostearic acid, with plausible relevance to Alzheimer’s disease (AD). Integrated network pharmacology and PPI analysis showed these metabolites converge on key AD hubs (ACHE, CHRM1, NOS1, NOS2) and related inflammatory-synaptic nodes, enriched in oxidative-stress response, cholinergic regulation, nitric oxide signaling, and arachidonic-acid metabolism.

Molecular docking demonstrated consistent moderate multi-target binding, strongest for unsaturated/oxygenated fatty acids, supporting a synergistic rather than single-target mode of action. In vitro assays further validated this pattern, revealing moderate antioxidant capacity and AChE/BChE inhibition compared with controls, aligning with the predicted mid-range affinities across several proteins. Overall, the findings indicate that *C. racemosa* extract may offer neuroprotective potential through combined cholinergic support, oxidative/nitrosative stress reduction, and anti-inflammatory lipid modulation appropriate for the multifactorial pathology of AD.

### 5.2. Recommendation

Future work should isolate and quantify the major bioactive lipids, confirm their mechanisms through enzyme-kinetic and target-validation assays (ACHE/BChE, NOS1/NOS2, and arachidonic-pathway enzymes), and evaluate efficacy in AD-relevant cellular and animal models while assessing BBB permeability, pharmacokinetics, safety, and synergistic interactions among metabolites to clarify therapeutic feasibility.

## 6. Patents

The authors disclose that the invention related to this work is covered by an Indonesian patent application S00202511597, filed by Universitas Islam Negeri Sunan Kalijaga Yogyakarta, with inventors Dr. Nita Handayani and Fahrul Nurkolis.

## Figures and Tables

**Table 1:** *Caulerpa racemosa* extract compounds (filtered from mzCloud best match > 95) via LC-HRMS.

Name	Formula	Calculated Molecular Weight	RT [min]	Area (Max.)	mzCloud Best Match
Hexadecanamide	C_16_H_33_NO	255.25505	21.71	132,989,235.55	98.6
Oleamide	C_18_H_35_NO	281.27056	21.54	104,636,468.21	98.3
Stearamide	C_18_H_37_NO	283.28606	23.77	82,837,237.90	97.3
α-Eleostearic acid	C_18_H_30_O_2_	278.22365	16.92	82,954,091.16	95.3
Palmitoleic acid	C_16_H_30_O_2_	254.22351	16.65	37,079,182.87	95.9
9-Oxo-10(E),12(E)-octadecadienoic acid	C_18_H_30_O_3_	294.21853	17.87	31,871,173.23	97.7
α-Linolenic acid	C_18_H_30_O_2_	278.22365	19.99	29,136,008.89	97.4
Palmitoyl ethanolamide	C_18_H_37_NO_2_	299.28127	20.29	16,462,133.15	97.3
2,4-dihydroxyheptadec-16-en-1-yl acetate	C_19_H_36_O_4_	350.24193	17.68	13,159,097.66	95.9

**Table 2:** The Vina score (kcal/mol) of the molecular docking parameter for the *Caulerpa racemosa* compounds against selected Alzheimer’s diseases protein.

Compounds	ACHE (PDB ID: 4EY7)	CHRM1 (PDB ID: 6WJC)	NOS2 (PDB ID: 1NSI)	NOS1 (PDB ID: 3NOS)
Hexadecanamide	−7.5	−6.5	−6.5	−6.0
Oleamide	−7.7	−6.9	−6.6	−6.4
Stearamide	−7.8	−6.5	−6.7	−6.1
α-Eleostearic acid	−8.3	−6.8	−7.5	−7.0
Palmitoleic acid	−7.6	−6.7	−6.8	−6.2
9-Oxo-10(E),12(E)-octadecadienoic acid	−8.1	−7.4	−7.3	−6.7
α-Linolenic acid	−8.4	−7.2	−7.3	−6.7
Palmitoyl ethanolamide	−7.5	−6.6	−6.7	−6.6
2,4-dihydroxyheptadec-16-en-1-yl acetate	−7.6	−6.6	−6.7	−6.2
Donepezil or Control	−11.8	−8.2	−10.7	−9.4

**Table 3:** Selected amino acid interaction of *Caulerpa racemosa* metabolites against Alzheimer’s diseases.

α-Linolenic acid vs. ACHE
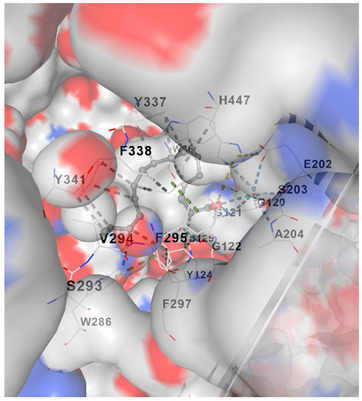 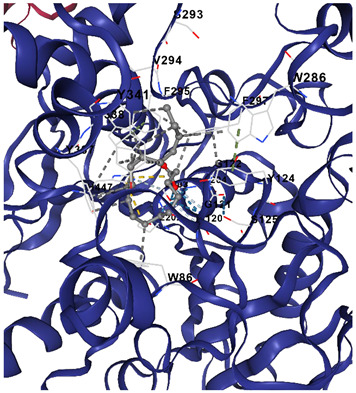
**Donepezil vs. ACHE**
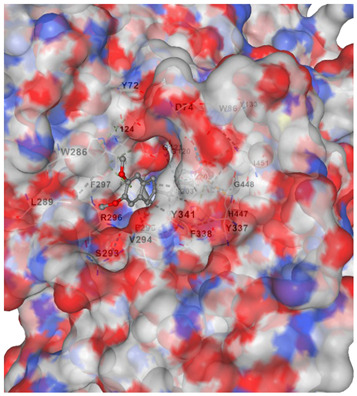 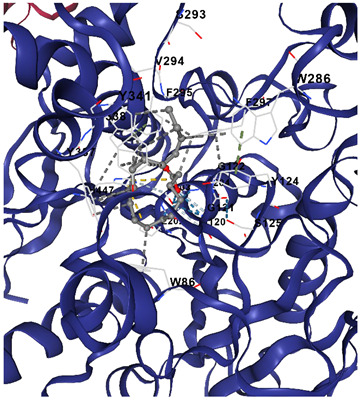

**Table 4:** IC_50_ (µg/mL) values of sea grape extract and controls.

No	Assay	Sample	Control	IC_50_ Extract (µg/mL)	IC_50_ Control (µg/mL)
1	DPPH Antioxidant	Sea grape extract	Trolox	120.97 ± 10.93 ^a^	99.04 ± 2.15 ^b^
2	AChE Inhibition	Sea grape extract	Physostigmine	136.48 ± 1.70 ^a^	82.34 ± 1.45 ^b^
3	BChE Inhibition	Sea grape extract	Physostigmine	145.98 ± 3.28 ^a^	83.81 ± 0.55 ^b^

Values are presented as mean ± SD (*n* = 3). Superscript letters “a” and “b” indicate statistically significant differences between the extract and control groups at *p* < 0.05.

## Data Availability

All original data and materials supporting the findings of this study are provided within the article and additional information or clarifications can be obtained by contacting the corresponding authors.
